# Efficient dibutyltin (DBT) elimination by the microscopic fungus *Metarhizium robertsii* under conditions of intensive aeration and ascorbic acid supplementation

**DOI:** 10.1007/s11356-017-8764-4

**Published:** 2017-03-27

**Authors:** Paulina Siewiera, Sylwia Różalska, Przemysław Bernat

**Affiliations:** Department of Industrial Microbiology and Biotechnology, Faculty of Biology and Environmental Protection, University of Lodz, Banacha 12/16, 90-237 Lodz, Poland

**Keywords:** *Metarhizium robertsii*, Dibutyltin utilization, Intense aeration, Hyphae morphology, Antioxidants, Oxidative stress alleviation, Malondialdehyde, Liquid chromatography

## Abstract

Dibutyltin (DBT) is an environmental pollutant characterized by immunotoxic, neurotoxic, and pro-oxidant properties. In this study, an attempt was made to enhance DBT elimination by the *Metarhizium robertsii* strain. We observed enhanced fungal growth in the bioreactor (pO_2_ ≥ 20%) compared to flask cultures (*μ*_max_ increased from 0.061 to 0.086 h^−1^). Moreover, under aerated conditions, *M. robertsii* mycelium with “hairy” morphology biodegraded DBT (20 mg l^−1^) 10-fold faster in the bioreactor than in the flask cultures. Monobutyltin (MBT) and a hydroxylated derivative of MBT (OHBuSnH_2_) were detected as by-products of dibutyltin debutylation. Simultaneous usage of glucose and butyltins indicates the comatabolic nature of monobutyltin and dibutyltin removal. In order to protect fungal cells from oxidative stress caused by DBT presence, vitamin C (20 mg l^−1^) was applied. Supplementation with ascorbic acid (AA) resulted in a 3-fold acceleration of MBT removal during the first 7 h of incubation. Using the HPLC-MS/MS technique, a quantitative analysis of malondialdehyde (MDA), a marker of oxidative stress, was performed. In the AA presence, a decrease in the MDA amount (about 45%) was observed compared to the case with fungal cells exposed to DBT alone.

**Electronic supplementary material:**

The online version of this article (doi:10.1007/s11356-017-8764-4) contains supplementary material, which is available to authorized users.

## Introduction

Dibutyltin (DBT) is a useful heat stabilizer of polyvinyl chloride, a curing agent for silicone rubbers, and a catalyst for esterification reactions. Because of its widespread use, the compound is found in the environment and in dietary sources (Moser et al. 2009). This organotin is mainly introduced into sediments and water by leaching from PVC materials. Additionally, dibutyltin is formed as a major degradation product of environmental tributyltin (TBT)—the most toxic of all organotin compounds. Due to its hydrophilicity, DBT rapidly enters into water and is accumulated in tissues of mussels and other marine invertebrates (Nesci et al. 2011). The concentrations of DBT in mussels from the Northern Adriatic Sea ranges from 15 to 2660 ng Sn g^−1^ (Nemanič et al. 2009). The levels of DBT in mussels collected from the Polish coast (Baltic Sea) are between 0.5 and 24 ng Sn g^−1^. On the other hand, dibutyltin found in the liver of European flounder in the Gdansk Bay represents 72–86% of total butyltin content (Albalat et al. 2002). The presence of DBT in human blood (4.7–36.7 ng Sn ml^−1^) and liver samples (0.4–12.8 ng Sn g^−1^) has been proved (Whalen et al. 1999; Nielsen and Strand 2002). Liver enzymes are not effective in DBT elimination (Albalat et al. 2002).

In contrast to tributyltin, the toxic effects of dibutyltin are less well known and there is little information available. A reduction of the toxicity of metabolites compared to the initial compound is assumed. Unfortunately, DBT is more immunotoxic to invertebrates and vertebrates than TBT (Frouin et al. 2008). Moreover, DBT has a stronger effect on mitochondrial functions than TBT, as it inhibits hydrolytic Mg-ATPase and Ca-ATPase activities in mussels (Bouchard et al. 1999; Nesci et al. 2011). A potential neurotoxic effect of DBT, which may lead to apoptotic death of the hippocampus and neocortex cells in rats, has also been reported (Jenkins et al. 2004). DBT is an inducer of oxidative stress and an amplifier of pro-inflammatory cytokine expression in microglia cells (Chantong et al. 2014).

In this study, an attempt was undertaken to improve the process of dibutyltin biodegradation using a microscopic fungus *Metarhizium robertsii.* The selected strain can eliminate TBT with high levels of efficiency by protecting the fungal cells from oxidative stress through the application of 17β-estradiol (Siewiera et al. 2015). In the present research, ascorbic acid (AA, vitamin C) and α-tocopherol (vitamin E) were chosen as primary antioxidants of the aqueous and lipophilic phases, respectively (Li and Schellhorn 2007). The effectiveness of vitamins in free radical scavenging was verified by quantitative analysis of malondialdehyde (MDA), a lipid peroxidation product. In order to speed up the process of DBT degradation, an additional oxygen supply (pO_2_ ≥ 20%) was prepared. Moreover, there was an attempt at the identification of metabolic intermediates formed during organotin dealkylation.

## Materials and methods

### Chemicals

Dibutyltin dichloride, ascorbic acid, α-tocopherol, methyl magnesium bromide, tropolone, anhydrous sodium sulfate, and 1,1,3,3-tetraethoxypropane were purchased from Sigma-Aldrich Chemical Co. (Germany). Stock solutions of DBTCl_2_, α-tocopherol (vitamin E), and ascorbic acid (vitamin C), each at a concentration of 10 mg ml^−1^, were prepared in ethanol, dimethyl sulfoxide, and distilled water, respectively. The solvents for organotin extraction such as methanol, hexane, and ethyl acetate were purchased from POCH S.A. (Poland). Other high purity organic solvents used during gas and liquid chromatography analyses originated from J.T. Baker Chemical Co. (the Netherlands).

### Microorganism and growth conditions

The ascomycete insect pathogenic fungus *M. robertsii* IM 6519 from the Department of Industrial Microbiology and Biotechnology (University of Lodz, Poland) was the subject of the study. The ability of the microorganism to degrade organotins was confirmed in an earlier paper (Siewiera et al. 2015).

Fourteen-day-old fungal cultures on ZT slants were used to inoculate synthetic medium (Lobos et al. 1992) in 100-ml Erlenmeyer flasks. The medium was modified and consisted of (grams per liter) K_2_HPO_4_ (4.36), KH_2_PO_4_ (1.7), MgSO_4_·7H_2_O (0.2), MnSO_4_ (0.05), FeSO_4_·7H_2_O (0.01), CaCl_2_·2H_2_O (0.03), glucose (40), yeast extract (10), and distilled water (up to 1 l), pH 6.8. The cultivation was carried out at 28 °C with shaking at 160 rpm for 24 h. The precultures were transferred to fresh medium (1:1 ratio) and incubated for another 24 h. In 100-ml flasks, the synthetic medium with DBT (20 mg l^−1^) or without the organotin (the control cultures) was inoculated with 20% of a homogeneous preculture. Incubation was conducted for 120 h in the above–mentioned conditions. Samples for analyses were collected after 0, 24, 48, 72, 96, and 120-h cultivations.

### Batch cultivations

Batch cultivations of the *M. robertsii* strain were conducted in a 3.6-l bioreactor (Labfors 5; Iris 6 software; Infors AG, Switzerland) with a culture volume of 1 l. The fungal preculture, obtained as described above, was additionally transferred to the fresh medium (1:2 ratio) and incubated for a further 24 h. Finally, the homogeneous preculture was introduced into 800 ml of the synthetic medium with DBT (20 mg l^−1^), either alone or in a mixture with one of the vitamins (C or E, both 20 mg l^−1^) or without the tested compounds (the control culture). The applied concentrations of the vitamins did not affect *M. robertsii* growth. The fungal cultures were incubated for 72 h with controlled aeration (air flow 1 l min^−1^), stirring (200–250 rpm), temperature (28 °C), and level of dissolved oxygen (pO_2_ ≥ 20%). The quantities of O_2_ in the introduced and exhaust gasses were measured with a gas analyzer (Infors AG, Switzerland). The pH of the medium was not regulated during the cultivation. In order to determine the fungal growth and DBT utilization, the samples were collected regularly: after 0, 3, 7, 12, 24, 48, and 72 h.

### Fungal biomass estimation

Fungal mycelia were separated from culture media by filtration through Whatman#1 filter paper and drying at 105 °C to reach a constant weight. The maximum specific growth rate (*μ*_max_) was calculated in accordance with the formula *μ*_max_ = [(ln*X*_2_ − ln*X*_1_) / (*t*_2_ − *t*_1_)], where *X*_2_ is the biomass concentration at time *t*_2_ and analogously for *X*_1_. Based on the growth curves, logarithmic growth phases of the *M. robertsii* strain were indicated. Mycelium amounts required for the calculations were taken from 0 and 24-h incubation and from 3 and 7-h cultivation for flask and batch cultures, respectively.

### Glucose content analysis

Glucose amounts in the supernatant were determined using an Agilent 1200 HPLC coupled with a QTRAP 3200 mass spectrometer (AB Sciex), according to the Bernat et al. (2013) procedure.

### Sample preparation and organotin determination

Acidified (pH 2) fungal biomass was suspended in methanol and homogenized with glass beads by ball milling (Retsch MM 400, Germany). After disruption, the samples were prepared according to the procedure by Bernat et al. (2013).

### The analysis of butyltin intermediates

The cultures (20 ml) were transferred into Falcon tubes and centrifuged for 10 min at 10,000*×g*. The mycelium was suspended in methanol and homogenized using a mixer mill with glass beads for 5 min at 30 m s^−1^. The extraction of the homogenate and supernatant mixture with ethyl acetate (1:1 *v*/*v*) was carried out twice. The organic layers were dehydrated with the use of anhydrous sodium sulfate and evaporated to dryness. The precipitate was dissolved in methanol.

Separation of butyltins was performed with the Agilent Technologies 1200 HPLC system equipped with a Phenomenex Aqua C18 125A column (50 mm × 2.0 mm × 5 μm) and maintained at 37 °C. The mobile phase consisted of water (A) and methanol (B), both supplemented with 2 mM ammonium formate and 0.2% formic acid. The run time was 9 min, and the solvent gradient was initiated at 60% B. After 1 min, the amount of B was increased to 100% over the following 2 min, and this was maintained for two additional minutes before returning to the initial solvent composition over the next 2 min, and this then being maintained for 2 min. The flow rate was 0.5 ml min^−1^ with an autosampler temperature of 10 °C and an injection volume of 10 μl, respectively.

To identify DBT derivatives in fungal samples by LC-MS/MS, an information-dependent acquisition (IDA) method was developed consisting of a precursor ion scan (PI) and an enhanced product ion (EPI) scan mode. IDA experiments were performed on a hybrid Q-Trap 3200 mass spectrometer (QTRAP; AB Sciex) connected to the HPLC system. The ion source conditions were set as follows: curtain gas (CUR) = 25, collision gas (CAD) = high, ionspray voltage (IS) = 5500, temperature (TEM) = 500, ion source gas 1 (GS1) = 40, and ion source gas 2 (GS2) = 50. Nitrogen was used as a nebulizer and an auxiliary gas. For the PI-EPI analysis, a PI scan of *m*/*z* 179 (BuSnH_2_^+^) was run in positive mode at a scan range from *m*/*z* 200 to *m*/*z* 650. The EPI scan was run in positive mode at a scan range for daughter ions from *m*/*z* 100 to *m*/*z* 700. Declustering potential (DP), entrance potential (EP), and collision energy (CE) were set to 25, 10, and 28, respectively.

### Characterization of morphological modifications

In order to determine the morphology of the *M. robertsii* hyphae from the exponential growth phase, image analysis was used. Fungal pellet morphology was performed for 30 pellets from each culture using the software package Axiovision 4.4 (Carl Zeiss, Germany). According to the Casas López et al. (2005) method, a central compact core region and a peripheral “hairy” region of the fungal pellets were separated. Subsequently, values of the pellet and pellet core projected area (mm^2^) were estimated.

### Quantitative determination of malondialdehyde

Samples were prepared according to the procedure by Wei et al. (2010) with some modifications. After 7 h of batch cultivation, 10 ml of the culture was collected and filtrated. The mycelium was washed with distilled water and transferred into Eppendorf tubes containing 1 ml of cooled water and glass beads. The homogenization process with the use of FastPrep 24 (MP Biomedicals, USA) was performed three times for 20 s at 4 m s^−1^ with 2-min breaks for cooling samples on ice. Subsequently, samples were centrifuged without beads for 10 min at 4000*×g* at 4 °C. The upper layers were transferred into inserts of dark glass vials for LC-MS/MS analysis. According to the Csallany et al. (1984) procedure, the MDA standard for quantitative determinations was obtained by acid hydrolysis of 1,1,3,3-tetraethoxypropane.

Measurement of MDA was performed using an Agilent 1200 HPLC (Santa Clara CA, USA) system and a 3200 QTRAP mass spectrometer (AB Sciex, Framingham, MA, USA) with an ESI source. For reversed-phase chromatographic analysis, 10 μl of the sample was injected into a Phenomenex Aqua C18 125A column (50 mm × 2.0 mm × 5 μm). The mobile phase consisted of water (A) and methanol (B); 5 mM ammonium formate was also used in all solvents as an additive. The solvent gradient was initiated at 20% B; after 0.5 min, this was increased to 100% B over 1 min and maintained at 100% B for four additional minutes before returning to the initial solvent composition over 2 min. The run time was 7 min. The column temperature was maintained at 37 °C, and the flow rate was 600 μl min^−1^. The instrumental settings were as follows: spray voltage −4500 V, curtain gas (CUR) 25, nebulizer gas (GS1) 55, turbo gas (GS2) 60, and ion source temperature of 500 °C. Data analysis was performed with Analyst™ v1.5.2 software (AB Sciex, Framingham, MA, USA). The monitored multiple reaction monitoring (MRM) pair for MDA was *m*/*z* 71–42.

### Statistical analysis

The experiments were carried out with triplicate samples. The Student’s *t* test and Spearman’s correlation were performed using Excel 2007 (Microsoft Corporation, USA). An average standard deviation (±SD) was calculated. Values were considered significant when *p* ≤ 0.05.

## Results and discussion

### Growth kinetics and DBT biotransformation by the *M. robertsii* strain incubated in flasks

**Fig. 1:**
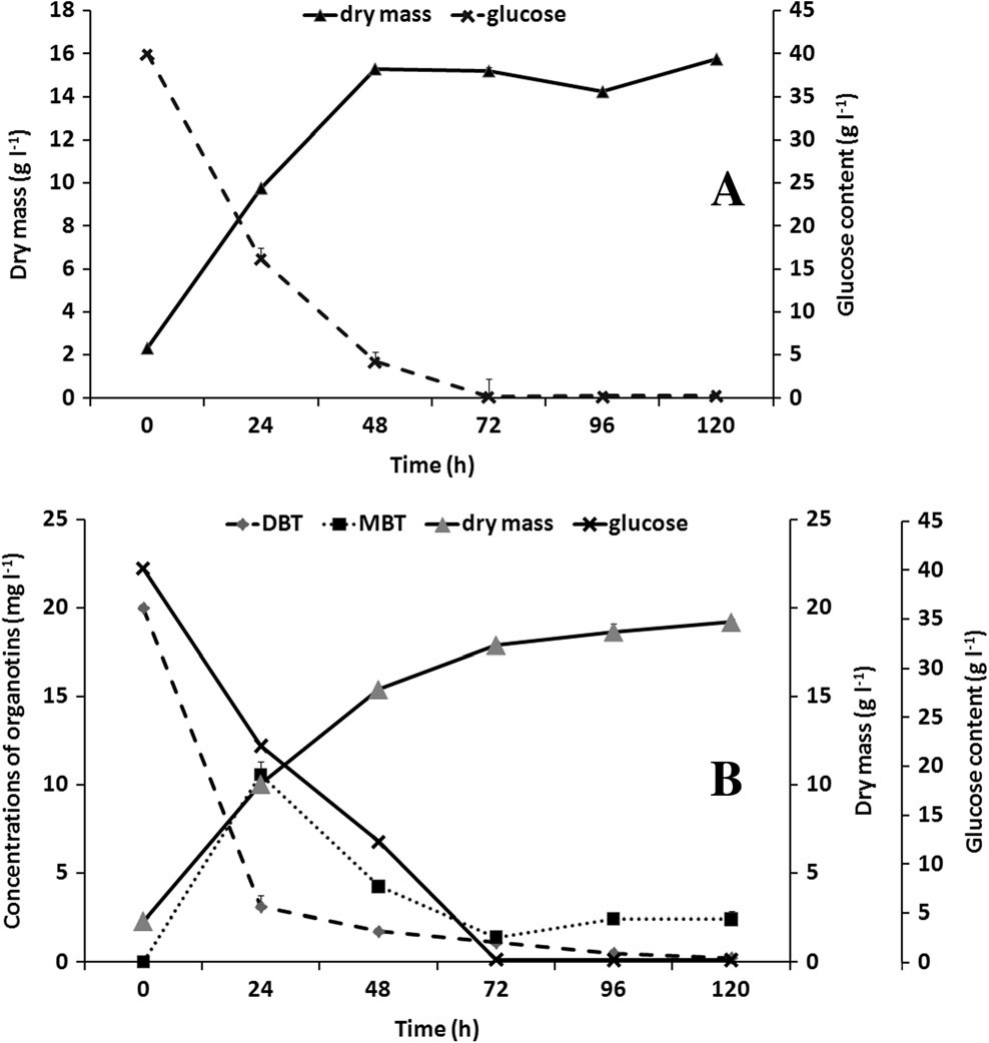
Biomass synthesis and glucose assimilation by the *M. robertsii* strain during flask cultivation without (**a**) and with an addition of DBT (**b**) for 120 h in synthetic medium. Additionally, in **b**, the curves for DBT and MBT biodegradation by the examined fungus incubated in the above conditions are shown

Based on the results of preliminary studies (data not shown), synthetic medium with an addition of yeast extract as a source of both organic nitrogen and various vitamins (Lee and Little 2015) was chosen for the *M. robertsii* strain cultivation. The growth kinetics and glucose assimilation by the fungal cells are shown in Fig. 1a. After 120-h incubation, the biomass amount was almost 16 g l^−1^, while the glucose uptake was complete after 72 h of cultivation. In the presence of DBT (20 mg l^−1^), an increase in the fungal dry weight of about 22% was observed (Fig. 1b). During the first 24 h of the experiment, glucose assimilation by those fungal cells exposed to the organotins was lower than in control cultures. At the same time, the most rapid decrease in DBT level was observed (Fig. 1b). No significant differences were noted in the substrate assimilation after 72 h of cultivation between the cultures, either with or without DBT. Due to the simultaneous utilization of glucose and the organotins, the cometabolic character of the compound removal was indicated. The same phenomenon has previously been described for DBT biodegradation by *Cochliobolus lunatus* and TBT elimination by both *Cunninghamella elegans* and *M. robertsii* (Bernat and Długoński 2006; Bernat et al. 2013; Siewiera et al. 2015).

The ability of the *M. robertsii* strain to eliminate not only dibutyltin but also its derivative compound—monobutyltin (MBT)—was confirmed. The efficiency of DBT removal after 5-day incubation on modified synthetic medium was estimated as 98%. On the other hand, a fifth of the MBT remained in the culture (Fig. 1b).

### Fungal growth and organotin utilization by the *M. robertsii* strain cultivated under intensive aeration conditions

**Fig. 2:**
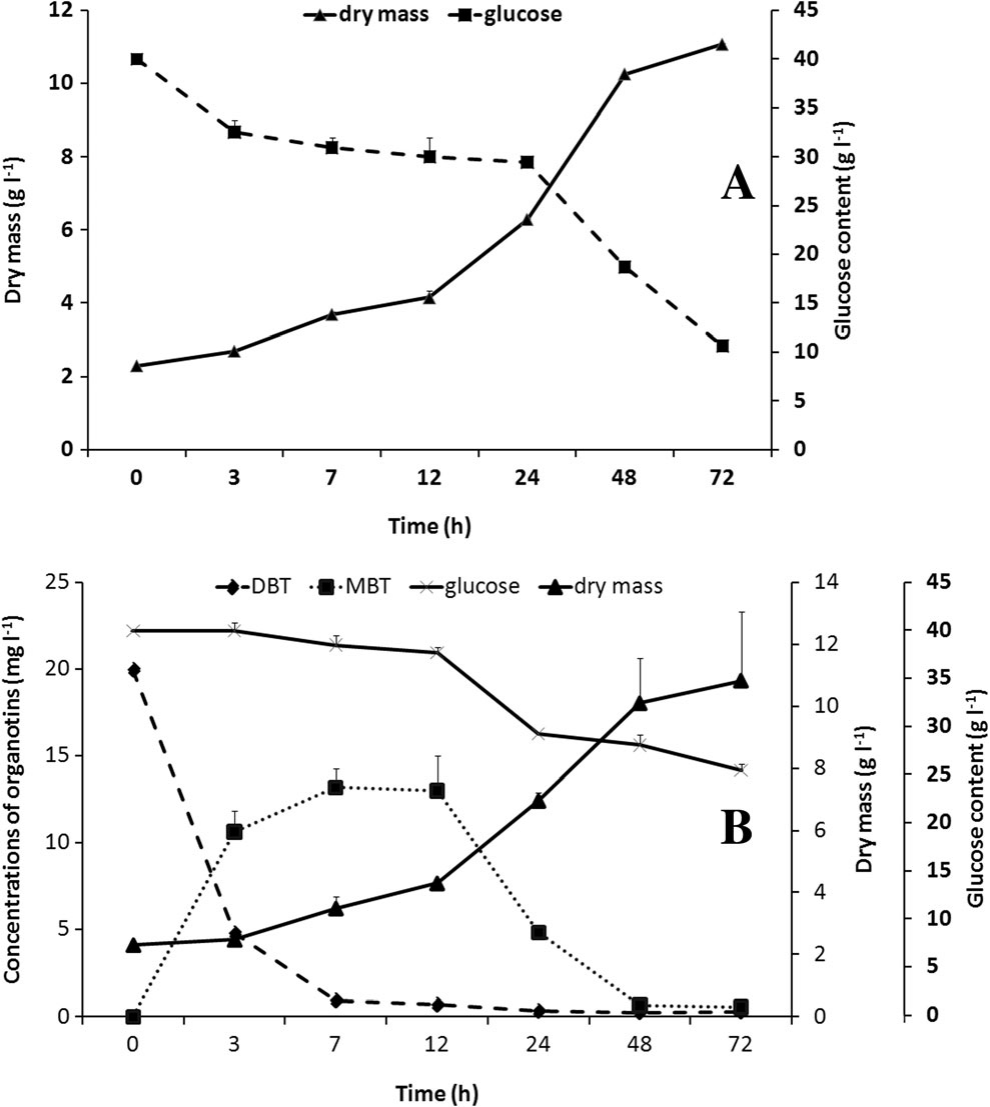
Biomass synthesis and glucose assimilation by the *M. robertsii* strain during batch cultivation without (**a**) and with an addition of DBT (**b**) for 72 h in synthetic medium. Additionally, in **b**, the curves for DBT and MBT biodegradation by the examined fungus incubated in the above conditions are shown

Although hyphae are not as sensitive as tissues (Bilodeau et al. 2005), mechanical agitation in a bioreactor undoubtedly causes stress resulting in morphological modifications in microorganism cells (Boswell et al. 2003; Chamsartra et al. 2005). However, the benefits of bioprocesses carried out on a bioreactor scale, such as culture homogeneity and facilitated transfers of nutrients, respiration gasses, and metabolic products (Garcia-Ochoa and Gomez 2009), seem to be more important than damage caused by mechanical stress. One positive impact of intensive oxygenation on fungal growth and organotin biodegradation has previously been discovered (Bernat and Długoński 2006). Therefore, in the next stage of the study, an experiment was performed with the aid of a bioreactor. After 72 h of batch cultivation, the fungal biomass from the control culture was lower (about 30% in comparison with the flask cultures) (Fig. 2a). Maximum specific growth rates (*μ*_max_) of the *M. robertsii* cells were also determined. The parameters for the fungus cultivated without DBT both in flask and batch cultures were 0.060 and 0.078 h^−1^, respectively. The acceleration of fungal growth in the bioreactor was caused by the efficient system supply of O_2_. Oxygen plays a key role in aerobic processes, especially in microorganism growth and metabolite production (Garcia-Ochoa and Gomez 2009). In our studies, the minimal level of dissolved oxygen was 20%. In the presence of DBT, pO_2_ = 20% was achieved after 17 h of incubation, 5 h later than in the fungal culture without the organotin. The changes in the productivity of the fungal biomass exposed to DBT were not significant (Fig. 2b), despite an increase in the *μ*_max_ value up to 0.086 h^−1^. A reverse dependency was observed in flask cultures supplemented with DBT. The maximum specific growth rates of the *M. robertsii* cells remained constant, despite the increase in the biomass amount compared to the control culture.

The rate of butyltin biotransformation by the fungal strain cultivated under intensive aeration conditions is presented in Fig. 2b. Although the final effectiveness of DBT elimination is comparable to the rate of the compound removal in flask cultures, a supply of oxygen resulted in a 10-fold acceleration of dibutyltin removal. A similar effect was described by Bernat and Długoński (2006) for TBT utilization by an additional oxygen supply to a growing *C. elegans* strain. The influence of the level of culture oxygenation on the progress of DBT debutylation by the *M. robertsii* is crucial. Moreover, complete metabolism of MBT by the fungus was possible only in the bioreactor culture. The positive effect of batch conditions on fungal growth and the organotin utilization by the microorganism is connected with a facilitated exchange of nutrients and respiratory gasses between the biomass and growth medium (Garcia-Ochoa and Gomez 2009).

In contrast to TBT, a compound biodegraded by bacteria (e.g., *Moraxella osloensis* (Yáñez et al. 2015), *Pseudomonas* sp. (Bernat et al. 2014), and *Enterobacter cloacae* (Sakultantimetha et al. 2011)), fungi (e.g., *C. elegans* (Bernat and Długoński 2006), *Cunninghamella echinulata* (Soboń et al. 2016), and *Coniophora puteana* (White et al. 1999)), the alga *Chlorella vulgaris* (Tsang et al. 1999), and crab *Thalamita crenata* (Chen et al. 2016), DBT and MBT have been described as being degraded by only a few microbial strains. Among the fungi, only *C. lunatus* has been mentioned as a strain efficiently degrading dibutyltin and monobutyltin. During incubation on Sabouraud medium, 92% of the initial DBT concentration (10 mg l^−1^) and approximately 70% of formed MBT were removed from the fungal culture after 24 and 168 h of cultivation, respectively (Bernat et al. 2013). On the other hand, the yield of DBT (20 mg l^−1^) transformation by *Streptomyce*s sp. was equal to 90% after 1-day cultivation on synthetic medium with a 2-fold higher amount of MBT in comparison to the *C. lunatus* strain. Moreover, within 7 days, almost 90% of produced MBT was removed from the bacterial culture (Bernat and Długoński 2009). The most significant advantage of our results, compared to those obtained in other published studies, is the reduction of the time required for the bioremediation process. During the first 24 h of cultivation, differences were slight, because all strains achieved a high (90%) efficiency of DBT elimination. However, MBT was still detected as a major by-product (until day 7), in both *Streptomyces* sp. (Bernat and Długoński 2009) and *C. lunatus* (Bernat et al. 2013) cultures, while as early as after 2 days of the *M. robertsii* cultivation, both butyltins were completely eliminated.

Unfortunately, no literature data concerning the comparison of dibutyltin elimination efficiency in flask and batch conditions are available. According to Moscoso et al. (2012), benzo[a]anthracene (BaA), one of the polycyclic aromatic hydrocarbons, is biodegraded by *Staphylococcus warneri* and *Bacillus pumilus* strains more efficiently in batch experiments than in flask conditions. After 2-day cultivation of the bacterial consortium on minimal medium with BaA (100 μM), 8 and 75% of initial compound concentration were removed in flask and bioreactor, respectively (Moscoso et al. 2012). On the other hand, in further studies on BaA biotransformation in the same conditions by the bacterial strain *Pseudomonas stutzeri*, a reverse dependency was observed. The efficiency of BaA utilization was equal to 94% in flasks and 81% in the bioreactor after 7 days of bacterial incubation (Moscoso et al. 2015). The published results indicate the importance of the metabolic abilities of microorganisms.

### The influence of vitamins C and E on DBT removal by the examined fungus

**Fig. 3:**
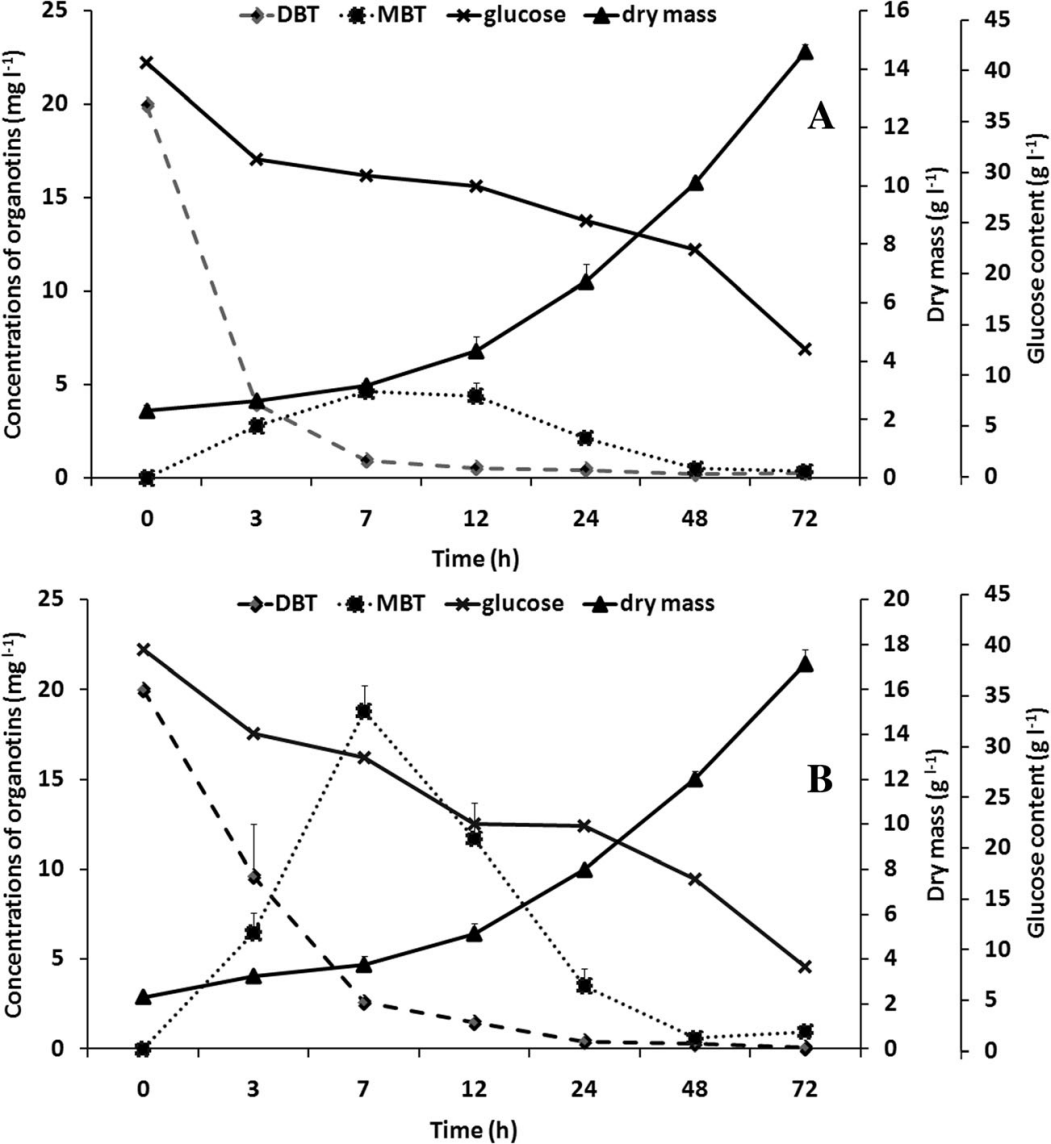
Growth curve, glucose assimilation, and butyltin utilization by the examined fungus incubated for 72 h in a bioreactor on synthetic medium supplemented with vitamin C (**a**) or vitamin E (**b**)

In our previous paper (Siewiera et al. 2015), an increase in the efficiency (about 14%) of TBT elimination by the *M. robertsii* strain cultivated in the presence of 17β-estradiol was confirmed. DBT, as well as TBT, promotes production of reactive oxygen species (ROS) (Chantong et al. 2014), which can inflict direct damage on cell components. In order to increase fungal tolerance to oxidative stress induced by the presence of DBT, vitamins C and E were applied. The influence of ascorbic acid (20 mg l^−1^) or α-tocopherol (20 mg l^−1^) on fungal biomass synthesis and organotin biodegradation is demonstrated in Fig. 3. In the presence of vitamins, the assimilation of glucose was about 50% higher for vitamin C (Fig. 3a) and about 68% higher for vitamin E (Fig. 3b) compared to culture with DBT alone. Moreover, biomass production increased about 34 and 58%, respectively. After supplementation of the growth medium with the tested vitamins, the values of *μ*_max_ were decreased compared to the control cultures and remained at the same level (~0.063 h^−1^). In the presence of antioxidants, faster oxygen uptake from growth medium by fungal cells was observed in comparison to the culture with DBT alone. pO_2_ = 20% was achieved after 14 and 9 h for vitamins C and E, respectively.

Vitamin supplementation played a greater role in MBT than in DBT elimination. The addition of ascorbic acid to the fungal culture led to a 3-fold acceleration of the rate of monobutyltin decomposition. The importance of vitamin C in heavy metal removal by the filamentous fungi was noted. According to Słaba et al. (2013), the addition of 1 mM ascorbic acid enhanced the uptake of lead and copper into the cell walls of *Paecilomyces marquandi*. In this study, in contrast to vitamin C, the negative impact of α-tocopherol on the MBT elimination was observed (about 40% during the first 7 h of incubation). The involvement of cytochrome P450 enzymes in vitamin E metabolism has been proved (Sontag and Parker 2002). Thus potentially, α-tocopherol could be a competitive inhibitor of CYP450, decreasing the rate of organotin debutylation. However, the involvement of the enzymatic complex in butyltin biodegradation by the *M. robertsii* strain has not yet been examined. On the other hand, the importance of vitamin E was confirmed in the biodegradation of polychlorobiphenyls as environmental pollutants. According to Ponce et al. (2011), α-tocopherol (1.6 μM) improved degradation of biphenyl and 4-chlorobiphenyl (4-CB) by the bacterial strain *Burkholderia xenovorans* cultivated in an aqueous solution. In the presence of the antioxidant, an increase in the rate of biphenyl degradation (40 μM) from 0.8 ± 0.1 to 1.32 ± 0.2 μM min^−1^ was observed. However, the efficiency of 4-CB (1 mM) elimination after 24-h incubation reached 40% in the absence of α-tocopherol and 100% in the presence of the antioxidant (Ponce et al. 2011).

In our experiments, butyltins were identified separately in the fungal biomass and the supernatant (data not shown). On this basis, the high affinity of DBT with the biomass was discovered. During the first hours of the study, as much as 69% of the initial concentration of the compound was attached to the *M. robertsii* mycelium. On the other hand, the location of the monobutyl derivative was dependent on the oxygen supply and incubation time. In the fungal cultures conducted in the bioreactor, continuous MBT excretion to the growth medium was detected. However, in the flask cultivation of the fungus, MBT was mostly transported to the substrate only for 24 h. Afterwards, accumulation of the monobutyl compound was observed in the hyphae.

A summary of the most important kinetic parameters of the *M. robertsii* growth and its efficiency in butyltin biodegradation is presented in Table 1, i.e., maximum values of biomass (*X*_max_), specific growth rate (*μ*_max_), DBT removal (%), MBT concentrations, and MBT removal (%). The regression coefficients (*R* ^2^) were higher than 0.98 in all cases, except for MBT removal (%) in the batch culture of the *M. robertsii* supplemented with DBT and vitamin E, where *R* ^2^ was equal to 0.877. The values of *R* ^2^ suggested at least good or very good fitting of the model, which confirmed its suitability for use in biodegradation processes.

**Table 1:** Kinetic parameters of *M. robertsii* growth and butyltin removal by the fungus cultivated on flask and bioreactor scale

Scale	Culture	Biomass parameters			Butyltin degradation parameters				
		*X*_max_ (g l^−1^)	*μ*_max_ (h^−1^)	*R* ^2^	Max DBT removal (%)	*R* ^2^	Max MBT concentration (mg l^−1^)	Max MBT removal (%)	*R* ^2^
Flasks	Control	15.75 ± 0.22	0.060	0.997	–	–	–	–	–
	DBT	19.20 ± 0.15	0.061	1.000	98.80 ± 0.15	0.997	10.57 ± 0.77	77.20 ± 2.10	0.987
	DBT + vitamin C	21.18 ± 0.87	0.064	0.999	94.65 ± 0.16	0.996	16.00 ± 0.17	67.25 ± 2.65	0.995
	DBT + vitamin E	22.00 ± 0.98	0.069	0.999	96.75 ± 0.21	0.986	11.07 ± 0.50	69.56 ± 2.17	0.989
Bioreactor	Control	11.07 ± 0.59	0.078	0.988	–	–	–	–	–
	DBT	10.83 ± 2.30	0.086	0.995	98.60 ± 0.10	0.999	13.20 ± 1.08	95.90 ± 0.90	0.984
	DBT + vitamin C	14.58 ± 0.28	0.063	1.000	98.60 ± 0.05	0.998	4.64 ± 0.20	92.46 ± 0.22	0.993
	DBT + vitamin E	17.15 ± 0.64	0.063	0.999	99.65 ± 0.20	0.996	18.78 ± 1.43	94.94 ± 0.05	0.877

*R* ^2^ coefficients refer to polynomial regression (*n* = 4)

Intensive aeration and the presence of vitamin C and glucose in medium ensured optimal conditions for fungal growth and butyltin biodegradation. The use of the nutrients is not cost-effective. However, the application of agricultural wastes as a rich source of carbon and energy for microorganisms (Singh and Nain 2014) is the first step in the reduction of costs with a simultaneous enhancement of biodegradation efficiency. Cometabolism could be a novel way of facilitating the removal of not only butyltins but also other pollutants.

### A qualitative analysis of butyltin metabolites

The extracts of the *M. robertsii* cultures from the exponential growth phase were chosen for the studies because of the rapid decrease in the DBT level at that time. The metabolites formed during the organotin biotransformation were qualitatively analyzed using gas and liquid chromatography coupled with mass spectrometry.

**Fig. 4:**
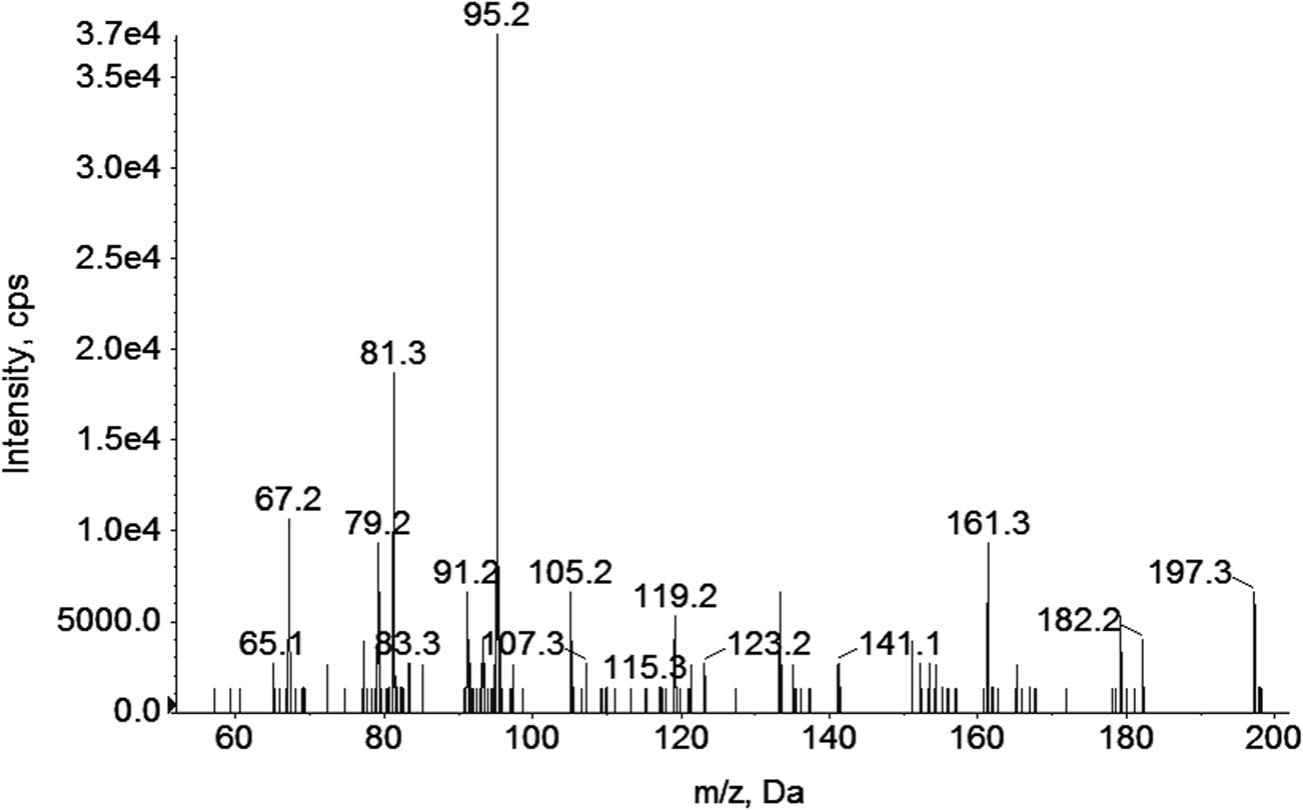
Mass spectrum showing the main ion group of OHBuSnH_2_ acquired on the first day of the *M. robertsii* incubation in the presence of DBT

**Fig. 5:**
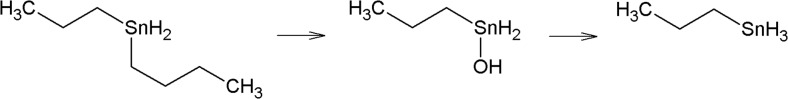
A proposed pathway of DBT biodegradation by the *M. robertsii *strain

MBT, the primary by-product of DBT biotransformation by the *M. robertsii* cells, was determined with GC-MS/MS. HPLC-MS/MS chromatograms revealed the presence of DBT and MBT and the formation of its metabolite at retention time 4.15 (Fig. 4). The retention times for DBT and MBT were 4.0 and 4.6, respectively. The spectrum of the analyte showed ions 197 and 179, and the appearance of the [SnH_3_]^+^ ion at *m*/*z* 123 and all of these exhibited a typical tin isotopic pattern. The difference in masses at 18 Da between 197 and 179 [BuSnH_2_]^+^ indicated that the obtained compound could be the hydroxylated derivative of MBT—OHBuSnH_2_. The hydroxylated derivative of DBT was determined in samples from the first to the third day of the *M. robertsii* incubation. Therefore, considering the obtained results, it seems that the presence of OHBuSnH_2_ was probably associated with DBT debutylation by the fungal strain (Fig. 5). A similar mechanism was described by Suzuki et al. (1992) and Matsuda et al. (1993) for TBT or DBT, which were metabolized either in vitro or in vivo by rat or fish liver microsome enzyme systems to MBT, DBT, and/or hydroxylated products at the third and fourth positions of DBT or TBT.

### Fungal cell morphology

Based on macroscopic observations during the experiments, the morphological modifications of the *M. robertsii* pellets (from the exponential growth phase) cultivated under various levels of oxygen supply were noted (Supplementary Fig. 1). Primarily, the shape and size of fungal morphological forms were examined. In the conditions of intensified oxygenation, the fungus grew as a non-spherical, dispersed form with long hyphae, while the fungus cultivated in flasks formed spherical, densely packed pellets with single hyphae outside the peripheral region. According to Papagianni’s (2004) hypothesis, hyphae aggregation and growth as pellets occur as a result of insufficient oxygen levels in the growth medium.

In order to characterize the *M. robertsii* cell morphology, the ratio between the pellet core and the projected area of the whole pellet was calculated (Table 2). A high ratio implied that the pellet had a “smooth” morphology while lower values of the ratio suggested a hairy morphology (Różalska et al. 2014). Densely packed fungal pellets with a smooth morphology were noted in the flask cultures, unlike the pellets from batch cultivation which had a large, actively growing hairy zone. Due to the exposure to DBT and supplementation with vitamins C or E, an increase in the hairy zone was observed. Moreover, there was a positive correlation (*r* = 0.803) between the degree of hyphae compaction and the efficiency of DBT biodegradation. Papagianni (2004) also reported that hyphae surrounding the pellet core are characterized by high activity. Taking into account the results obtained for the *M. robertsii* during butyltin and 4-n-nonylphenol biotransformation (Różalska et al. 2014), hyphae compaction seems to be a common feature of the xenobiotic metabolism by fungi from the genus *Metarhizium*.

**Table 2:** The ratio between the pellet core and the projected area of the whole pellet of the *M. robertsii* strain cultivated in flasks and in a bioreactor on synthetic medium in the absence of DBT, in the presence of DBT alone, or in a mixture with one of the vitamins

	Control	DBT	DBT + vitamin C	DBT + vitamin E
Cultures in flasks	0.732 ± 0.076	0.950 ± 0.029	n.t.	n.t.
Cultures in bioreactor	0.430 ± 0.020	0.562 ± 0.006	0.629 ± 0.048	0.536 ± 0.090

*n.t.* not tested

### Analysis of lipid peroxidation products

Dibutyltin contributes indirectly to lipid oxidation by inducing reactive oxygen species (Chantong et al. 2014). MDA is the most mutagenic among secondary products formed during the process of lipid peroxidation (Ayala et al. 2014). In this study, quantitative analyses of MDA, one of the most popular and reliable markers of oxidative stress, were conducted with the use of liquid chromatography coupled with mass spectrometry. Extracts of the *M. robertsii* batch cultures exposed to DBT (with or without the antioxidants) from the exponential growth phase were examined. The highest level of MDA, i.e., 151 μM l^−1^, was determined for those fungal cells supplemented with the organotin alone. In the presence of ascorbic acid and α-tocopherol, a decrease in the MDA amount was observed, about 45 and 2%, respectively. The same effect was characterized by Lu et al. (2007), who investigated pancreatic damage in rats induced by the presence of dibutyltin dichloride. The action of AA, determined as soothing, was supported by the measurement of reduced MDA levels after the antioxidant treatment (Lu et al. 2007). In order to detect MDA in the organs of rabbits exposed to stannous chloride (the ROS inducer), a reaction with thiobarbituric acid was applied. Researchers have reported that treatment with ascorbic acid causes a decrease in the TBARS levels in all tested organs (El-Demerdash et al. 2005). These findings are consistent with our results, despite the use of different test organisms.

Undoubtedly, the reduction of MDA quantity in fungal membranes damaged by DBT was a result of the efficient process of free radical scavenging by ascorbic acid. Consequently, restriction of oxidative stress was the main reason for the improved yield of the process of DBT biodegradation by the examined fungus.

## Conclusions

This report is the first to show that the *M. robertsii* can degrade both DBT and MBT with high levels of efficiency. An additional supply of oxygen led to a hairy morphology for the *M. robertsii* hyphae instead of densely interwoven pellets. A facilitated exchange of nutrients and respiratory gasses between the biomass and growth medium contributed to intensive fungal growth and finally improved butyltin biodegradation. Due to simultaneous utilization of glucose and the organotins, the cometabolic character of the described process is suggested. Moreover, supplementation of the growth medium with ascorbic acid protects fungal cells exposed to DBT from oxidative stress.

## Electronic supplementary material

Supplementary Fig. 1 (DOCX 3798 kb).
