# Supplementary material for: Efficient dibutyltin (DBT) elimination by the microscopic fungus Metarhizium robertsii under conditions of intensive aeration and ascorbic acid supplementation
Source: Environ Sci Pollut Res Int. 2017 Mar 27;24(13):12118–27. doi: 10.1007/s11356-017-8764-4 (PMC5410213; doi:10.1007/s11356-017-8764-4)

Supplementary: Fig.1. Confocal micrographs of mycelium structures from flask (A, B) and bioreactor cultures (C, D) conducted on synthetic medium supplemented with DBT (B, D) or without the compound (A, C). Microscope magnification was 10x. The scale bar represents 100 µm.


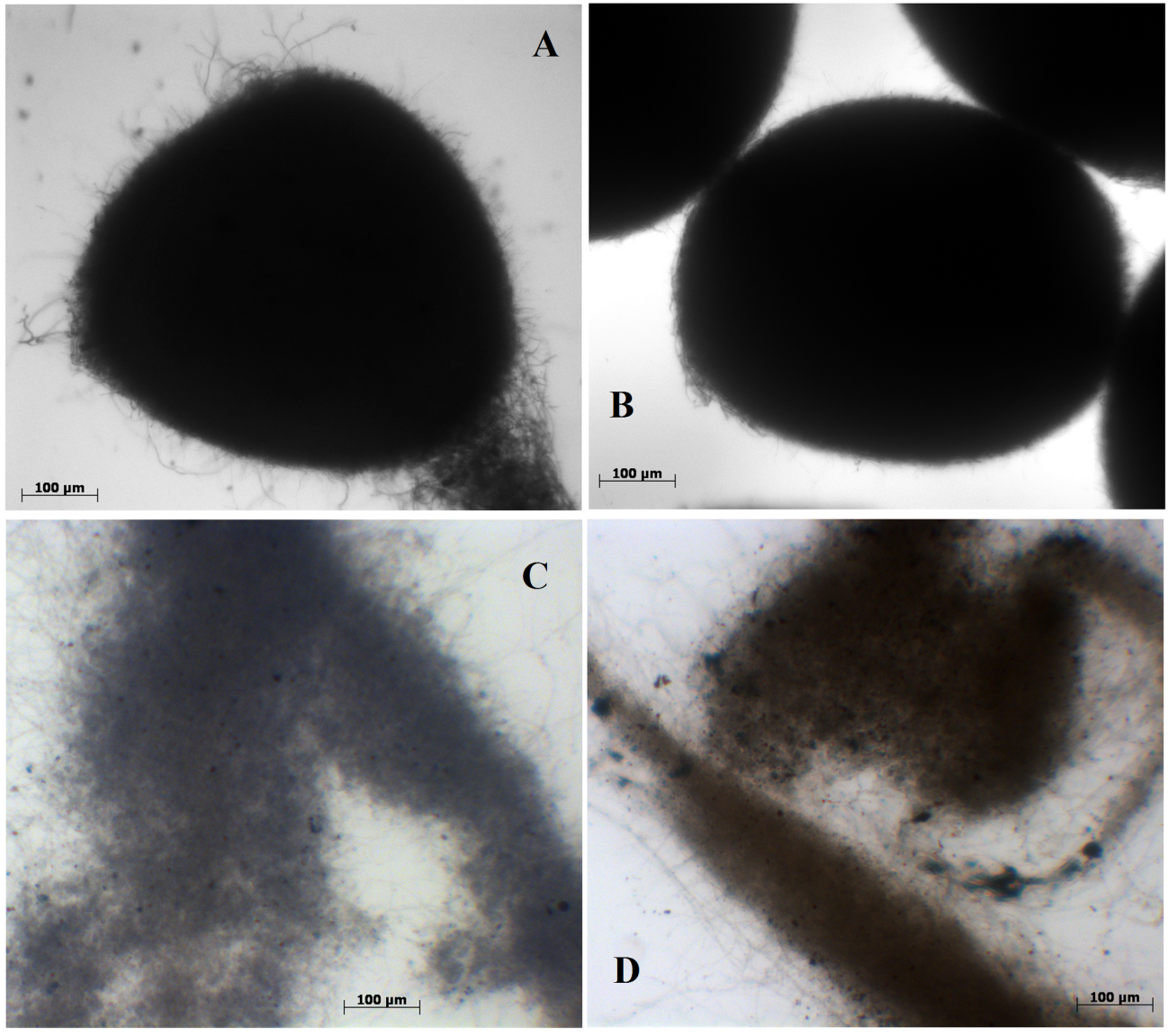

Supplement: Supplementary file 1 — (DOCX 3798 kb). [file 11356_2017_8764_MOESM1_ESM.docx]
